# Authors’ response: Oxygen therapy in heart failure

**DOI:** 10.1093/ehjopen/oeaf122

**Published:** 2025-09-30

**Authors:** Maxime Tremblay-Gravel, Jacinthe Boulet, Jean-Claude Tardif

**Affiliations:** Montreal Heart Institute, Université de Montréal, 5000 Bélanger Est, Montreal, Quebec H1T 1C8, Canada; Montreal Heart Institute, Université de Montréal, 5000 Bélanger Est, Montreal, Quebec H1T 1C8, Canada; Montreal Heart Institute, Université de Montréal, 5000 Bélanger Est, Montreal, Quebec H1T 1C8, Canada; Montreal Health Innovations Coordinating Centre, Montreal Heart Institute, Université de Montréal, 5000 Bélanger Est, Montreal, Quebec H1T 1C8, Canada

**Keywords:** Exercise, Heart failure, Oxygen, Quality of life, Pulmonary hypertension

**This correspondence refers to ‘Oxygen supplementation in ambulatory patients with heart failure: a randomized proof-of-concept study’, by M. Tremblay-Gravel *et al.* https://doi.org/10.1093/ehjopen/oeaf074.**

**Authors’ response to the commentary of Kourek and Dimopoulos, https://doi.org/10.1093/ehjopen/oeaf121.**

We thank Kourek and Dimopoulos for their thoughtful commentary^1^ on our proof-of-concept study^2^ evaluating ambulatory oxygen therapy in patients with chronic heart failure (HF). We agree that the methodological and physiological considerations they raise are important, and we welcome the opportunity to clarify several points.

First, we acknowledge the concerns regarding potential deleterious haemodynamic effects of hyperoxia. Experimental data suggest that high inspired oxygen concentrations can impair cardiac performance. However, whether these acute physiological changes translate into adverse clinical outcomes in chronic HF remains uncertain, leaving significant equipoise. We also note that the cited pilot work by Dimopoulos *et al*.^3^ was conducted in pulmonary arterial hypertension rather than HF, a distinct population in which extrapolation is not straightforward. This adds to the uncertainty and highlights the need for HF-specific evidence.

Second, patient heterogeneity is another consideration. While some of our participants had a left ventricular ejection fraction above 40%, our study was not powered for subgroup analyses and its small sample size precludes meaningful segmentation of the population. More importantly, the field of HF is moving beyond a rigid ejection fraction–based framework, as several key therapies, including angiotensin receptor–neprilysin inhibitors, mineralocorticoid receptor antagonists, and SGLT2 inhibitors, have demonstrated benefits across the ejection fraction spectrum.^4^ All of our study participants shared the same hallmark symptoms of HF, namely, exertional dyspnoea and reduced functional capacity corroborated by elevated N-terminal pro-B-type natriuretic peptide, which were the focus of this exploratory trial.

We also agree with the importance of disentangling the cardiac and pulmonary contributions to breathlessness. Although one patient had chronic obstructive pulmonary disease in our study, comorbid pulmonary disease is common in HF, and future studies should carefully characterize pulmonary function with spirometry in all patients. This is particularly relevant given that oxygen therapy has established benefits in chronic obstructive pulmonary disease and related conditions.^5^

Finally, we concur that in advanced or refractory HF, priorities may shift towards symptom relief even at the expense of potential physiological trade-offs. Patients sometimes place a higher value on quality of life than on longevity, and this perspective should inform the design and goals of future trials.^6^ It is essential that future clinical trials clearly define whether the aim is to improve HF outcomes or to provide palliative benefit. That said, there is not yet definitive evidence that oxygen therapy confers symptomatic benefit in HF. Our study was small, unblinded, and exploratory, and its results should be considered only as hypothesis-generating.

In summary, we emphasize that our findings should not be extrapolated to routine clinical practice at this stage. Rather, they should stimulate the conduct of the larger, rigorous, and well-stratified studies that are needed to explore the role of ambulatory oxygen therapy in HF.
